# *CHST9* rs1436904 genetic variant contributes to prognosis of triple-negative breast cancer

**DOI:** 10.1038/s41598-017-12306-6

**Published:** 2017-09-18

**Authors:** Jupeng Yuan, Nasha Zhang, Hui Zhu, Jibing Liu, Huaixin Xing, Fei Ma, Ming Yang

**Affiliations:** 1 Shandong Provincial Key Laboratory of Radiation Oncology, Cancer Research Center, Shandong Cancer Hospital affiliated to Shandong University, Shandong Academy of Medical Sciences, Jinan, Shandong Province China; 2 Department of Radiation Oncology, Shandong Cancer Hospital affiliated to Shandong University, Shandong Academy of Medical Sciences, Jinan, Shandong Province China; 3 Department of Medical Oncology, Cancer Hospital, Chinese Academy of Medical Sciences, Beijing, China

## Abstract

Triple-negative breast cancer (TNBC) refers to one aggressive histological subtype of breast cancer with high heterogeneity and poor prognosis after standard therapy. Lack of clearly established molecular mechanism driving TNBC progression makes personalized therapy more difficult. Thus, identification of genetic variants associated with TNBC prognosis will show clinic significance for individualized treatments. Our study is aimed to evaluate the prognostic value of the genome wide association study (GWAS)-identified *CHST9* rs1436904 and *AQP4* rs527616 genetic variants in our established early-stage TNBC sample database. Cox regression was used to estimate hazard ratios (HRs) and 95% confidence intervals (CIs). *CHST9* rs1436904G allele was significantly associated with decreased disease-free survival time (DFS) (8.5 months shorter in GG genotype carriers compared to TT genotype carriers, HR = 1.70, 95% CI = 1.03–2.81, *P* = 0.038). Stratified analyses showed an increased risk of cancer progression in *CHST9* rs1436904G allele carriers harboring larger tumor (tumor size > 2 cm), without lymph-node metastasis, being premenopausal at diagnosis or with vascular invasion (*P* = 0.032, 0.017, 0.008 or 0.003). Our findings demonstrate that the GWAS-identified 18q11.2 *CHST9* rs1436904 polymorphism significantly contributes to prognosis of early-stage TNBC, suggesting its clinical potential in the screening of high-risk TNBC patients for recurrence and the possibility of patient-tailored therapeutic decisions.

## Introduction

Breast cancer is one of the most common malignancies worldwide, with an estimated 255,180 new cases and 41,070 deaths in the United States in 2017^1^. Notably, the incidence and mortality of breast cancer have increased tremendously in developing countries including China. Breast cancer is responsible for 15% of all new Chinese women cancer patients^2^. With similar carcinoma genetic mechanisms to other kinds of solid tumor, development of breast cancer is a chronic and multiple-step process involving accumulation of genetic and epigenetic alterations^3,4^. Risk factors for breast cancer include obesity, lack of physical exercise, alcohol abuse, hormone replacement therapy during menopause, ionizing radiation, early age at first menstruation, having children late or not at all, older age, and family history^3–9^. Triple-negative breast cancer (TNBC) refers to one type of breast cancer that does not express estrogen receptor (ER), progesterone receptor (PR) and Her2/neu^10–14^. TNBC patients usually have relatively poor outcomes due to its intrinsically aggressive behaviors and requires combination therapies instead of common chemotherapies because loss of target receptors. Thus, more effective and sensitive prognostic markers are instantly needed to guide clinical management of TNBC precisely.

Many genetic variants have been described to contribute to breast cancer risk since the discovery of *BRCA1* and *BRCA2* in 1990s^15–22^. These genetic inheritable variants are associated with familial breast cancer. Thus, identifying new genetic variants will show great significance in breast cancer prediction and treatments. Recent genome-wide analyses based on large consortia do avoid false positive identification of candidate genes^23^. Two 18q11.2 genetic variants (*CHST9* rs1436904 and *AQP4* rs527616) were identified as novel breast cancer susceptibility components based on GWAS^24^. One nested case-control study based on female Chinese patients within Singapore Chinese Health Study were also performed to verify the roles of these SNPs. It has been demonstrated that genetic variants on top of conventional risk factors did improve the risk prediction of breast cancer in Chinese women^25^, but not clear enough to declare whether *CHST9* rs1436904 and *AQP4* rs527616 affect prognosis of TNBC. To test this, we conducted a hospital-based cohort study of early-stage TNBC to further illustrate the role of these two genetic variants in breast cancer progress. We found that the *CHST9* rs1436904 polymorphism might be a potential prognostic biomarker for early-stage TNBC, especially in the patients harboring larger tumor (tumor size > 2 cm), without lymph-node metastasis, being premenopausal at diagnosis or with vascular invasion.

## Materials and Methods

### Study subjects

A total of 381 TNBC patients were recruited between January 2008 and December 2015 at Cancer Hospital, Chinese Academy of Medical Sciences (Beijing, China). These patients were followed until May 6, 2016 in order to collect data on clinicopathological characteristics, treatments, and vital status, such as recurrence and death. Disease free survival (DFS) was defined as the time from the date of diagnosis until the date of the first locoregional recurrence, first distant metastasis, or death due to any cause. Patients known to be alive with no evidences of disease progression were censored at the last follow-up date or on May 6, 2016 (whichever came first). All subjects were ethnic Han Chinese. As we can see from histopathological data, most majority of recruited TNBC patients were at early-stage. At recruitment, the informed consent was obtained from each subject. This study was approved by the institutional Review Boards of Cancer Hospital, Chinese Academy of Medical Sciences and Shandong Cancer Hospital affiliated to Shandong University.

Immunohistochemistry (IHC) of formalin-fixed, paraffin-embedded breast cancer tissue samples obtained from the patients was used to evaluate ER or PR status with anti-ER and anti-PR antibodies. A positive ER or PR status was defined by nuclear staining of more than 1% based on guidelines of American Society of Clinical Oncology (ASCO) and College of American Pathologists (CAP) in 2010. To determine the HER2 status, IHC or gene amplification was performed by fluorescence *in situ* hybridization (FISH). Tumors negative for ER, PR, and HER2 were defined as TNBCs.

### Genotyping

A total of 1 mL blood sample was collected from each patient upon recruitment. Genomic DNA was extracted from the blood. The *CHST9* rs1436904 and *AQP4* rs527616 polymorphisms were analyzed by the MassArray system (Sequenom Inc., San Diego, California, USA) as described previously^26–29^. *CHST9* rs1436904 PCR primers are 5′-ACGTTGGATGCTTCCCTGCAAGACTATGTG-3′ (Forward) and 5′-ACGTTGGATGGCAAGACAGGAGACAGATTC-3′ (Reverse). *CHST9* rs1436904 UEP_SEQ primer is 5′-CCCCCTTGTGTCTCATTCCTCA-3′. *CHST9* rs1436904 EXT1_SEQ primer is 5′-CCCCCTTGTGTCTCATTCCTCAG-3′. *CHST9* rs1436904 EXT2_SEQ primer is 5′-CCCCCTTGTGTCTCATTCCTCAT-3′. *AQP4* rs527616 PCR primers are 5′-ACGTTGGATGTTACACGAGACTGAGCCAAC-3′ (Forward) and 5′-ACGTTGGATGGAAATGCCCCTTAGGACAAG-3′ (Reverse). *AQP4* rs527616 UEP_SEQ primer is 5′-GAGCTCCAGTGCTATTT-3′. *AQP4* rs527616 EXT1_SEQ primer is 5′-GAGCTCCAGTGCTATTTC-3′. *AQP4* rs527616 EXT2_SEQ primer is 5′-GAGCTCCAGTGCTATTTG-3′. A 20% blind, random sample of study subjects was genotyped in duplicates and the reproducibility was 100%.

### Statistics

The differences of patient clinical characteristics were calculated using Student’s *t* test or χ^2^ test. DFS was calculated as the time to progression or death without progression from the date of diagnosis. Survival distributions were estimated with the Kaplan-Meier method and were compared using log-rank test. The multivariate Cox proportional hazards model was applied to estimate effects of prognostic factors on DFS, using proverbial clinical factors, including age of onset, body mass index (BMI), tumor size, lymph-node metastasis, histological type, histological grade, menopausal status, vascular invasion, breast or ovarian cancer history, surgical method, taxane/anthracycline-based chemotherapy and radiotherapy, where it was appropriate. References for multivariate analyses were without family history of breast cancer or ovarian cancer for breast cancer or ovarian cancer history, postmenopausal at diagnosis for menopausal status at diagnosis, modified radical mastectomy for operation method,histological grade I for histological grade, without vascular invasion for vascular invasion, tumor size ≤ 2 cm for tumor size, without lymph-node involvement for lymph-node involvement, without acceptance of chemotherapy for taxane/anthracycline-based chemotherapy, and without acceptance of radiotherapy for radiotherapy. *P* value of less than 0.05 was used as the criterion of statistical significance. All statistical procedures were conducted using SPSS software (version 16.0).

## Results

### TNBC patients’ characteristics and clinical outcomes

A total of 381 TNBC patients were enrolled in this study. All individuals were female ethnic Han Chinese. The distribution of demographic and clinical characteristics of patients were showed in Supplementary Table 1. By the time of the final analysis (May 2016), the median follow-up time of the patients was 45.5 months. One hundred and eighty-two patients (49.2%) had disease progression and the median DFS time was 36.0 months (range, 0–204 months).

### Comparison of survival according to baseline characteristics of TNBC patients

To test whether various clinical characteristics contribute to DFS, patients were grouped according to age of onset, BMI, tumor size, lymph-node metastasis, histological type, histological grade, menopausal status, vascular invasion, breast or ovarian cancer history, surgical method, taxane/anthracycline-based chemotherapy and radiotherapy, respectively. DFS was compared between (or among) different sub-groups. As shown in Supplementary Table 2, BMI, histological grade, vascular invasion, lymph-node metastasis and radiotherapy can significantly influence DFS independently (*P* < 0.05). However, other baseline characteristics did not affect DFS (*P* > 0.05). After adjustments for other clinicopathologic factors, only BMI and vascular invasion showed statistically significant impacts on patient prognosis (Supplementary Table 2).

### Effects of *CHST9* rs1436904 and *AQP4* rs527616 polymorphisms on TNBC DFS

**Figure 1:**
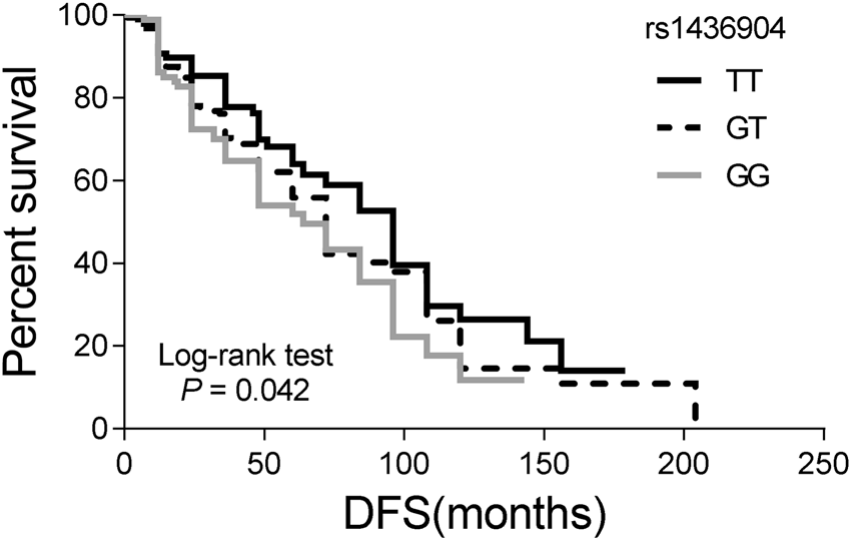
Kaplan-Meier curves of DFS for TNBC patients harboring different genotypes of *CHST9* rs1436904 genetic variations. Survival curves of different genotypes were compared by Kaplan-Meier method, followed by long-rank test (*P* = 0.042).

It has been demonstrated that *CHST9* rs1436904 and *AQP4* rs527616 are breast cancer susceptibility single nucleotide polymorphisms (SNPs)^24,25^. However, the role of those two genetic variations in TNBC patients’ outcome has not been examined. Genotype frequencies of *CHST9* rs1436904 and *AQP4* rs527616 SNPs among patients were summarized in Table 1. Interestingly, only *CHST9* rs1436904 polymorphism was significantly associated with DFS of TNBC patients. The mean DFS of TNBC patients with the *CHST9* rs1436904 GG genotype (46.8 months) or the GT genotype (50.1 months) was significantly shorter than that of the TT group (55.3 months). Moreover, both univariate and multivariate Cox proportional hazards model indicated that the *CHST9* rs1436904 genetic variation was significantly associated with disease progression of TNBC patients (Table 1 and Fig. 1). After adjustments of multiple clinical factors, the *CHST9* rs1436904 GG genotype was still significantly associated with disease progression compared to subjects with the TT genotype (HR = 1.70, 95% CI = 1.03–2.81, *P* = 0.038). Similarly, the risk of early recurrence for TNBC patients carrying the *CHST9* rs1436904 G allele (GT and GG genotype) increased about 1.51-folds (95% CI = 1.03–2.22) in comparison with TT genotype patients (*P* = 0.033).

**Table 1:** Genotype frequencies of rs1436904 and rs527616 polymorphism among TNBC patients and their association with DFS.

SNPs	Genotype	Patients No. (%)	DFS (month) Mean (25th, 75th)	Univariate analysis		Multivariate analysis	
				HR (95% CI)	*P*	HR (95% CI)	*P*
rs1436904		378					
	TT	97(25.7)	55.3(30, 68)	Reference		Reference	
	GT	194(51.3)	50.1(24, 65)	1.31(0.91–1.88)	0.151	1.42(0.95–2.13)	0.086
	GG	87(23.0)	46.8(24, 62)	1.58(1.03–2.41)	0.036	1.70(1.03–2.81)	0.038
	GT + GG	281(74.3)	49.1(24, 64)	1.38(0.98–1.95)	0.069	1.51(1.03–2.22)	0.033
rs527616		375					
	GG	167(44.5)	48.1(25, 62)	Reference		Reference	
	GC	179(47.8)	54.1(30, 71)	0.84(0.62–1.14)	0.256	0.78(0.56–1.09)	0.143
	CC	29(7.7)	48.9(24, 61)	1.06(0.62–1.82)	0.828	1.21(0.62–2.38)	0.582
	GC + CC	208(55.5)	53.4(27, 70)	0.87(0.65–1.16)	0.332	0.81(0.59–1.12)	0.211

Note: DFS, disease–free survival time; HR, hazard ratio; CI, confidence interval.

Hazard ratios (HRs) and 95% confidence intervals (CIs) for the association between SNP and disease-free survival time (DFS) were estimated by Cox regression adjusted by age of onset, BMI, tumor size, lymph-node involvement, histological type, histological grade, menopausal status, vascular invasion, breast or ovarian cancer history, surgical method, taxane/anthracycline-based chemotherapy and radiotherapy.

### Stratified analyses of the effects *CHST9* rs1436904 on DFS of TNBC patients

The association between *CHST9* rs1436904 polymorphism and DFS of TNBC patients was further examined by stratifying for age of onset, BMI, tumor size, lymph-node metastasis, histological type, histological grade, menopausal status, vascular invasion, breast or ovarian cancer history, surgical method, taxane/anthracycline-based chemotherapy and radiotherapy, respectively (Table 2 and Supplementary Table 3–7).

**Table 2:** DFS of TNBC associated with *CHST9* rs1436904 genotypes by tumor size, lymph-node involvement, menopausal status as well as vascular invasion

Variable		*CHST9* rs1436904				
		Genotypes	Patients No. (%)	DFS (month) Mean(25th, 75th)	HR (95% CI)	*P*
Tumor size	≤ 2 cm		179			
		TT	46(25.7)	54.9(32, 72)	Reference	
		GT	90(50.3)	53.2(29, 67)	1.11(0.61–2.02)	0.726
		GG	43(24.0)	47.7(24, 69)	1.36(0.62–2.99)	0.444
		GT + GG	133(74.3)	51.5(27, 69)	1.19(0.69–2.06)	0.534
	> 2 cm		195			
		TT	50(25.6)	55.9(28, 64)	Reference	
		GT	102(52.3)	46.3(24, 62)	1.61(0.88–2.95)	0.122
		GG	43(22.1)	46.4(24, 60)	2.16(0.97–4.81)	0.060
		GT + GG	145(74.4)	46.4(24, 62)	1.88(1.06–3.35)	0.032
Lymph-node involvement	No		229			
		TT	58(25.3)	62.6(36, 72)	Reference	
		GT	118(51.5)	53.9(31, 72)	1.85(1.01–3.37)	0.046
		GG	53(23.2)	48.1(26, 70)	2.27(1.07–4.83)	0.033
		GT + GG	171(74.7)	52.1(30, 71)	2.01(1.13–3.57)	0.017
	Yes		142			
		TT	38(26.8)	44.5(15, 55)	Reference	
		GT	73(51.4)	42.7(20, 57)	1.15(0.61–2.16)	0.675
		GG	31(21.8)	45.3(24, 60)	1.39(0.60–3.23)	0.450
		GT + GG	104(73.2)	43.5(20, 58)	1.29(0.72–2.32)	0.390
Menopausal status at diagnosis	Premenopausal		179			
		TT	46(25.7)	48.0(36, 83)	Reference	
		GT	94(52.5)	48.0(27, 62)	2.07(1.18–3.65)	0.011
		GG	39(21.8)	36.0(32, 64)	2.18(1.12–4.22)	0.021
		GT + GG	133(74.3)	45.0(27, 62)	2.10(1.22–3.63)	0.008
	Postmenopausal		39			
		TT	46(28.8)	43.0(26, 61)	Reference	
		GT	76(47.5)	51.5(28, 71)	0.96(0.51–1.82)	0.904
		GG	38(23.8)	40.5(30, 63)	1.41(0.68–2.93)	0.360
		GT + GG	114(71.2)	48.0(29, 71)	1.09(0.60–1.97)	0.779
Vascular invasion	No		334			
		TT	85(25.4)	48.0(31, 64)	Reference	
		GT	174(52.1)	48.0(27, 65)	1.29(0.85–1.95)	0.227
		GG	75(22.5)	43.0(32, 67)	1.19(0.73–1.95)	0.484
		GT + GG	249(74.6)	48.0(27, 65)	1.26(0.85–1.87)	0.252
	Yes		39			
		TT	14(35.9)	41.0(27, 92)	Reference	
		GT	16(41.0)	40.0(12, 60)	3.71(0.99–13.94)	0.052
		GG	9(23.1)	18.0(12, 24)	39.37(7.27–213.22)	<0.001
		GT + GG	25(64.1)	24.0(12, 57)	6.51(1.89–22.36)	0.003

Note: DFS, disease–free survival time; HR, hazard ratio; CI, confidence interval.

Hazard ratios (HRs) and 95% confidence intervals (CIs) for the association between SNP and disease-free survival time (DFS) were estimated by Cox regression adjusted by age of onset, BMI, tumor size, lymph-node involvement, histological type, histological grade, menopausal status, vascular invasion, breast or ovarian cancer history, surgical method, taxane/anthracycline-based chemotherapy and radiotherapy.

**Figure 2:**
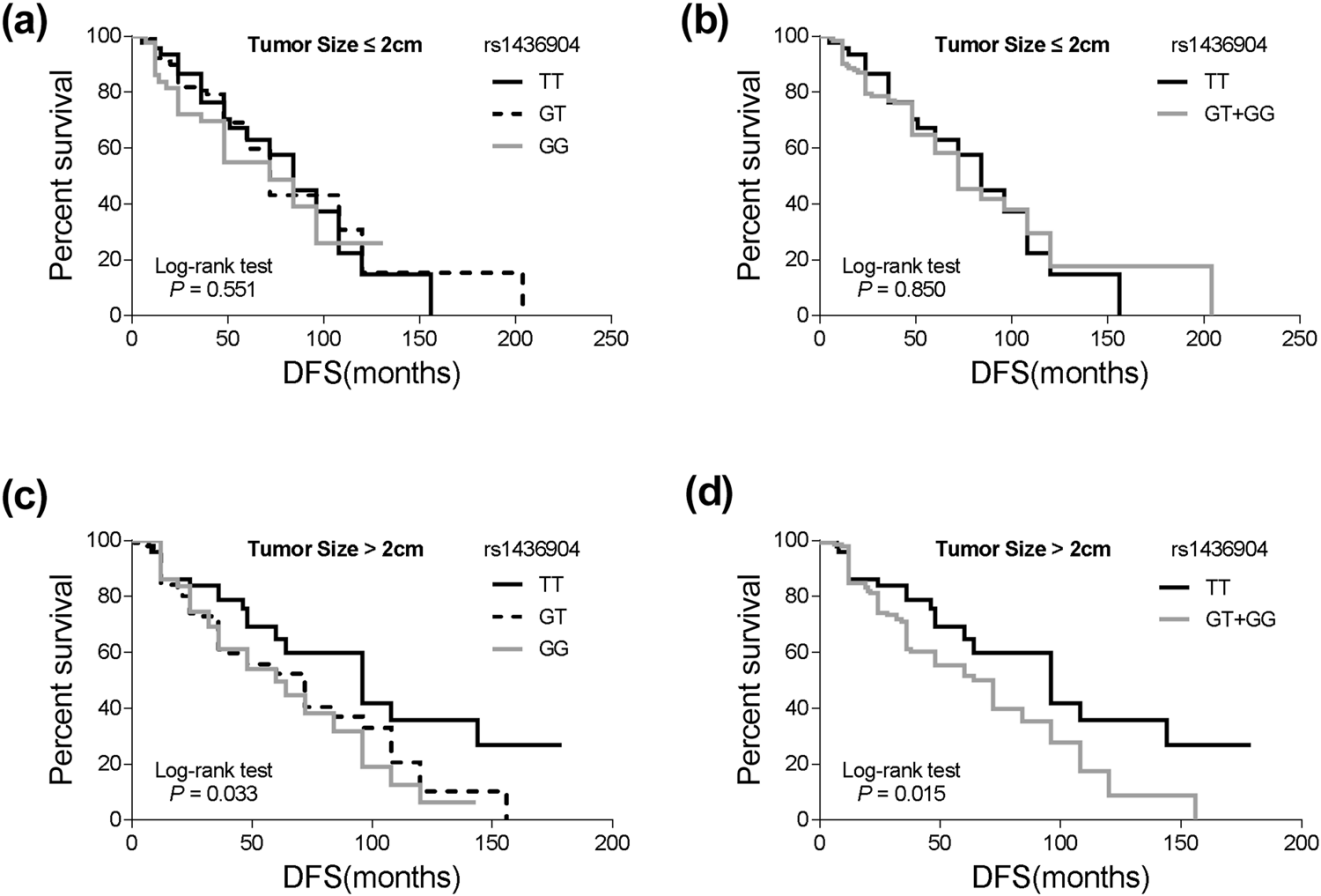
Stratified analyses of DFS for TNBC patients with different genotypes of *CHST9* rs1436904 genetic variations according to tumor size. (**a**,**b**) DFS for the patients harboring small tumors (≤2 cm; *P* = 0.551 or 0.850 for different classifications); (**c**,**d**) DFS for the patients harboring large tumors (>2 cm; *P* = 0.033 or 0.015 for different classifications).

In the subgroup of TNBC patients harboring large tumors (tumor size > 2 cm), the *CHST9* rs1436904 G allele (GT and GG genotype) was associated with a significantly increased risk of disease progression (HR = 1.88, 95% CI = 1.06–3.35; *P* = 0.032) compared to the TT genotype. The mean DFS of the G allele carriers was obviously shorter compared to the cases with the TT genotype (46.4 months vs. 55.9 months; *P* = 0.015) (Table 2 and Fig. 2). However, such differences were not observed in patients with small tumors (tumor size ≤ 2 cm), indicating that the *CHST9* rs1436904 polymorphism was an independent prognostic marker of TNBC cases with large tumors.

**Figure 3:**
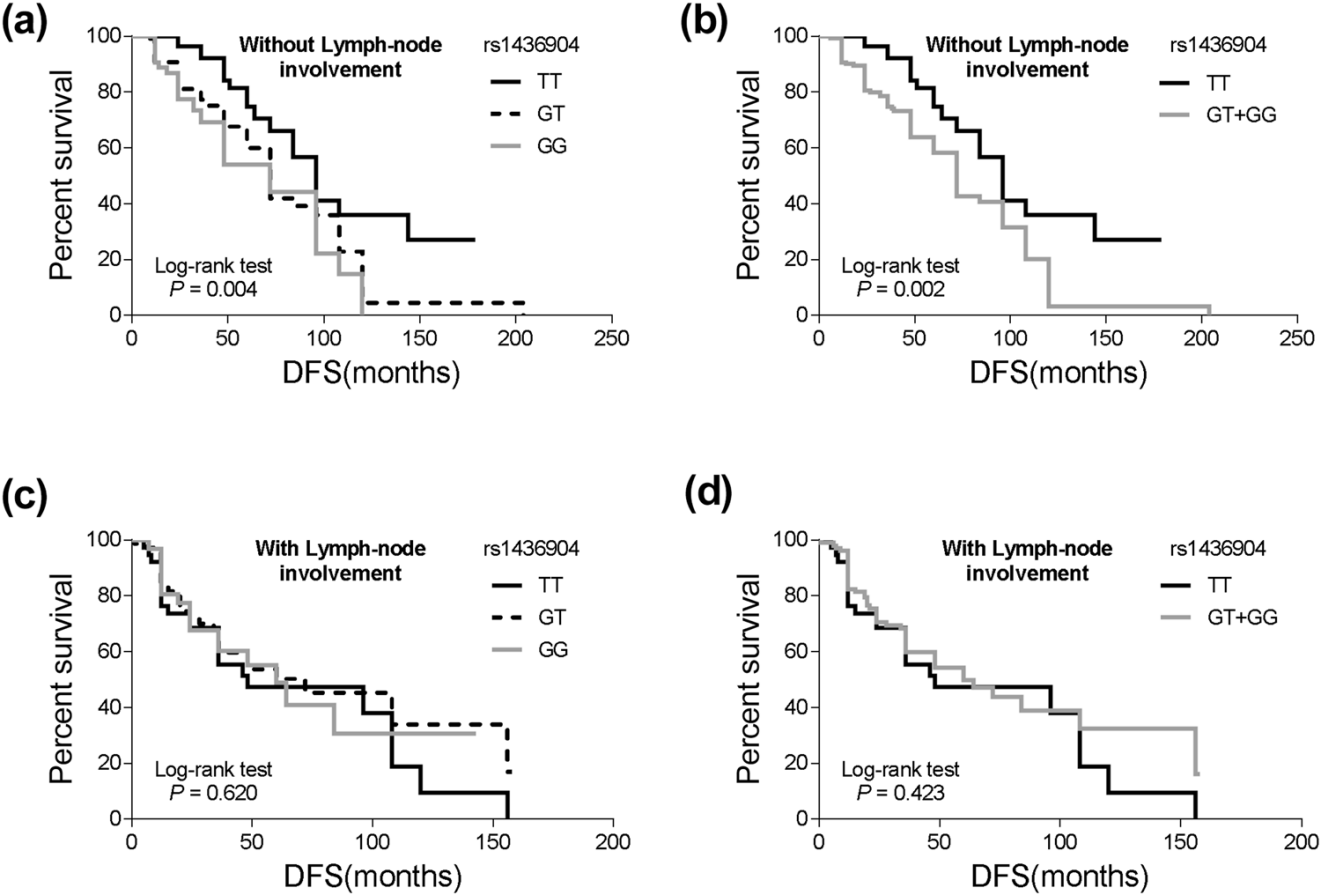
Stratified analyses of DFS for TNBC patients with different genotypes of *CHST9* rs1436904 genetic variations according to lymph-node metastasis. (**a**,**b**) DFS for the patients without lymph-node metastasis (*P* = 0.004 and 0.002 for different grouping mode); (**c**,**d**) DFS for the patients with lymph-node metastasis (*P* = 0.620 and 0.423 for different grouping mode).

Among the TNBC patients without lymph-node metastasis, the mean DFS of the *CHST9* rs1436904 GG genotype was significantly shorter than that of the TT genotype patients (48.1 months vs. 62.6 months; *P* = 0.033) (Table 2 and Fig. 3). However, there was no such association between the polymorphism and DFS in patients with lymph-node metastasis (mean DFS of the TT, GT and GG genotypes: 48 months, 72 months and 60 months, respectively; *P* = 0.620). HRs, calculated from the multivariate Cox proportional hazards model, demonstrated that patients without lymph-node metastasis harboring *CHST9* rs1436904 GG or GT/GG genotype showed 2.27-fold or 2.01-fold increased risk for disease progression (*P* = 0.033 or 0.017, respectively) compared to the TT genotype patients (Table 2).

**Figure 4:**
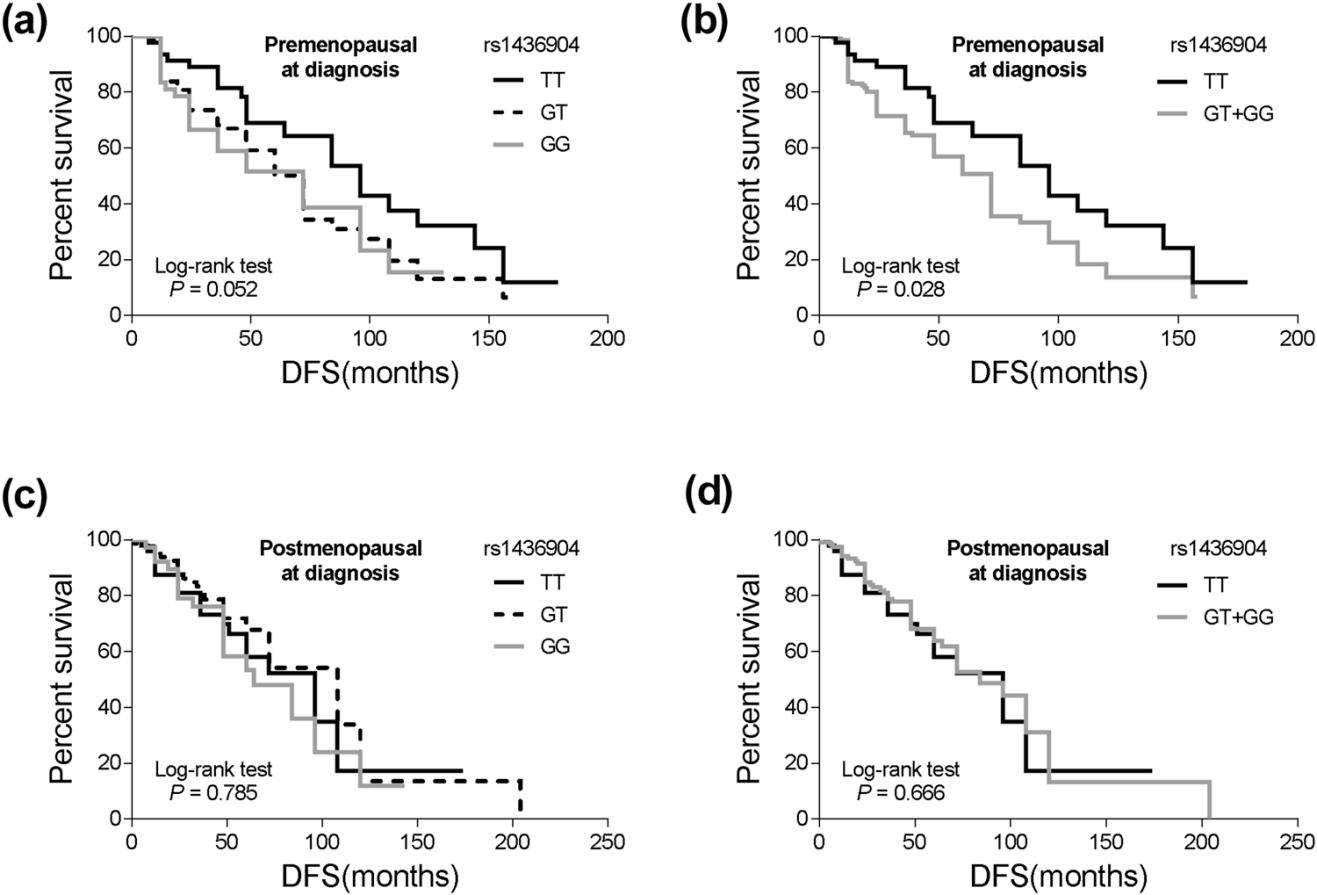
Stratified analyses of DFS for TNBC patients with different genotypes of *CHST9* rs1436904 genetic variations according to menstrual status. (**a**,**b**) DFS for the patients being premenopausalat at diagnosis (*P* = 0.052 and 0.028 for different grouping mode); (**c**,**d**) DFS for the patients being postmenopausalat at diagnosis (*P* = 0.785 and 0.666 for different grouping mode).

Among the patients who were premenopausal at diagnosis, the median DFS of either the rs1436904 GT or GG genotype patients (48.0 months or 36.0 months) was shorter than that of the rs1436904 TT genotype patients (48.0 months). However, there was no such association between the polymorphism and DFS in patients being postmenopausal at diagnosis (Table 2 and Fig. 4). In the multivariate Cox proportional hazards model, premenopausal patients with the rs1436904 GG genotype showed 2.18-fold increased risk for disease progression (95% CI = 1.12–4.22, *P* = 0.021) compared to subjects with the TT genotype (Table 2). Similar results were observed among premenopausal patients with the rs1436904 GT genotype (HR = 2.07, 95% CI = 1.18–3.65, *P* = 0.011) (Table 2).

**Figure 5:**
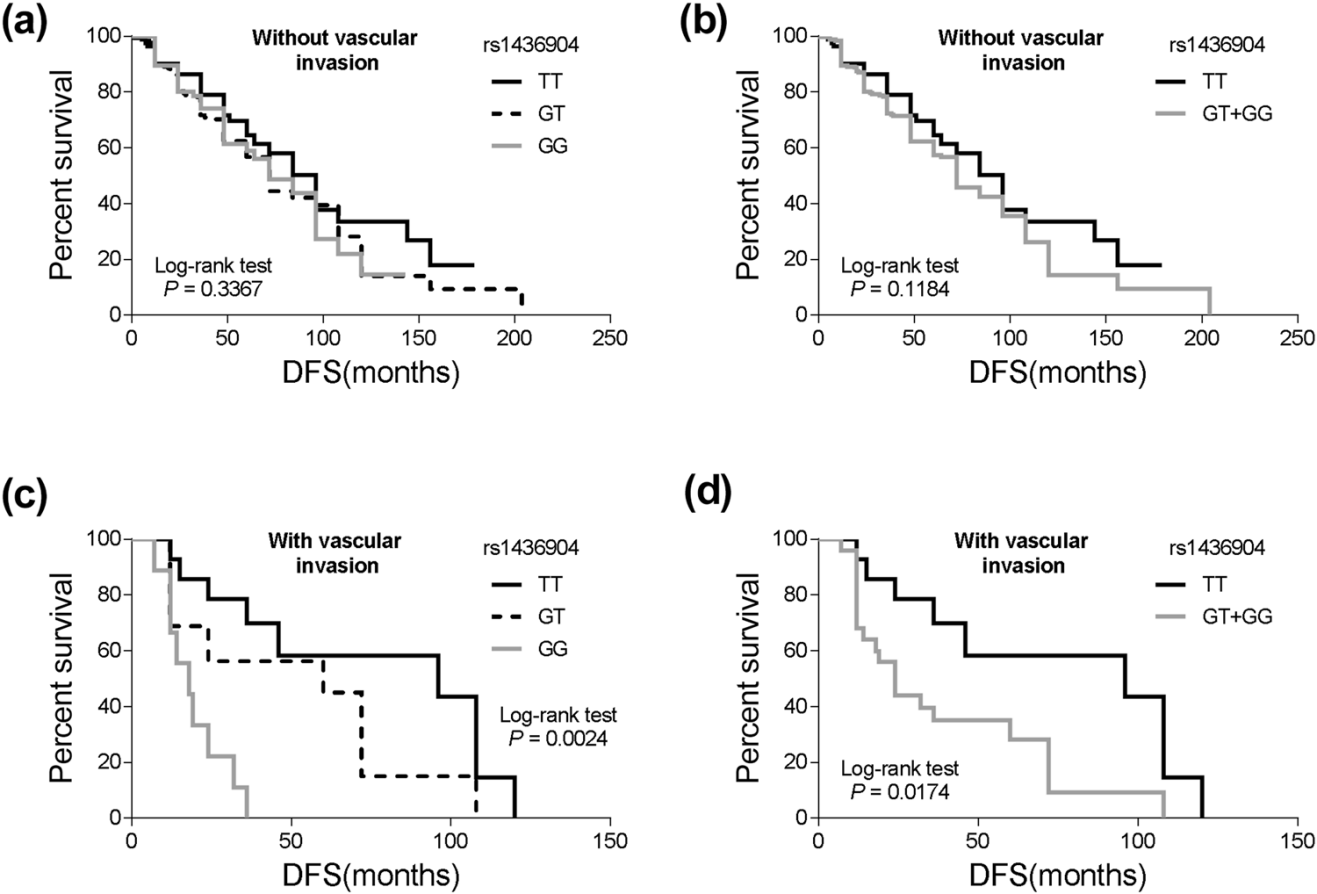
Stratified analyses of DFS for TNBC patients with different genotypes of *CHST9* rs1436904 genetic variations according to vascular invasion. (**a**,**b**) DFS for the patients being premenopausalat at diagnosis (*P* = 0.337 and 0.1184 for different grouping mode); (**c**,**d**) DFS for the patients being postmenopausalat at diagnosis (*P* = 0.0024 and 0.0174 for different grouping mode).

In the subgroup of TNBC patients with vascular invasion, the *CHST9* rs1436904 G allele was significantly associated with an increased risk of disease progression compared to the TT genotype (HR = 6.51, 95% CI = 1.89–22.36; *P* = 0.003). Especially, TNBC patients with GG genotype showed 39.37-fold increase risk for disease progression (*P* < 0.001). However, such differences were not observed in patients without vascular invasion (Table 2 and Fig. 5).

## Discussions

Development of breast cancer are multiple-process consequences of combined genetic and epigenetic changes^3,4^. About five to ten percent of breast cancer cases are believed to be hereditary and associated with certain gene mutations^20–22^. Although multiple breast cancer susceptibility genes have been identified, new sets of susceptibility genes should also be identified. TNBC accounts for 12–24% of breast cancers associating with early recurrence and poor outcome. Additional efforts should be made to discover specific loci or genetic variants related to TNBC risk, which will expand our understanding of the etiology of this aggressive breast cancer and improve its prevention and clinical diagnosis. Currently, genome-widely analysis was performed to discover and validate genetic variants that are associated with breast cancer risk using large consortia^15–18^. Multiple novel breast cancer genetic susceptibility loci were identified and validated base on this approach^24^. Based on a breast cancer GWAS, which identified *AQP4* rs527616 and *CHST9* rs1436904 genetic variants, we explored their involvement in early-stage TNBC and concluded that the *CHST9* rs1436904 SNP is an independent prognostic genetic variant in Chinese TNBC patients. These results will provide new prevention and diagnosis targets in TNBC therapy.

In Michailidou *et al*.’s study^24^, the G (minor allele) in relative to T (major allele) is a relative “protective” risk against breast cancer (OR = 0.96, 95% CI = 0.94–0.98). However, we did observe the significant role of the same SNP on prognosis of TNBC. There might be multiple reasons. First of all, the purpose of our study is to identify prognostic markers, but the GWAS study was aimed to identify susceptibility SNPs. As a result, the observation of different results due to different study purpose might be possible. For example, *ERCC1* C118T was associated with lung cancer risk. The OR was 0.90 (95% CI = 0.81–0.99, *P* = 0.043) in an additive genetic model (C allele vs. T allele) and 0.77 (95% CI: 0.63–0.95, *P* = 0.013) in a recessive genetic model (CC/CT vs. TT)^30^. However, *ERCC1* C118T was proved to be a risk SNP of overall survival for platinum-based chemotherapy in Asian NSCLC patients (CT + TT versus CC: HR = 1.24, 95% CI = 1.01–1.53)^31^.

The two SNPs examined in this study locate in *AQP4* and *CHST9* gene. *AQP4* belongs to AQP family and functions in water maintaining and ion homeostasis^32^. It is located at membrane and cytoplasmic fraction and markedly decreased in tumor tissues compared to paired-adjacent tissue, thus indicating its pathogenic role during cancer development^33^. CHST9 belongs to the N-acetylgalactosamine 4 sulfotransferase (GalNAc4ST) family, which transfers sulfate to position 4 of nonreducing terminal GalNAc residues^34^. Sulfate group I carbohydrates play important roles in conferring highly specific functions on glycoproteins, glycolipids, and proteoglycans^35^. It plays an important role in hematologic malignancies because *CHST9* copy number variants (CNV) are associated with acute myelogenous leukemia (AML)^36^. Additionally, *CHST9* CNV and amplification are also found in the brain of schizophrenia patients and gastric cancer patients with metastatic lymph node^37,38^. Nevertheless, the role of CHST9 as well as its genetic variations in breast cancer, especially TNBC, has not been determined. Our results show that *CHST9* rs1436904 SNP significantly contributes to early-stage TNBC progression risk. Notably, our results also show that the *CHST9* rs1436904 G allele is a “risk” genetic variant for outcome of TNBC patients, significantly associated with shorten DFS in TNBC patients harboring big tumors (>2 cm), without metastasis, being premenopausal at diagnosis or with vascular invasion.

In all, to the best of our knowledge, our study for the first time identified an inherited variation in *CHST9* which was significantly associated with DFS of TNBC patients, especially in TNBC patients harboring big tumors, without lymph-node metastasis, being premenopausal at diagnosis or with vascular invasion. Our findings might have potential clinical implications on precision treatment of TNBC, and eventually affect the therapeutic efficacy.

### Ethical approval

All procedures performed in studies involving human participants were in accordance with the ethical standards of the institutional and/or national research committee and with the 1964 Helsinki declaration and its later amendments or comparable ethical standards.

## Electronic supplementary material

Supplementary tables
