# Supplementary material for: CHST9 rs1436904 genetic variant contributes to prognosis of triple-negative breast cancer
Source: Sci Rep. 2017 Sep 18;7:11802. doi: 10.1038/s41598-017-12306-6 (PMC5603563; doi:10.1038/s41598-017-12306-6)
Supplement: Supplementary file 1 — Supplementary tables [file 41598_2017_12306_MOESM1_ESM.doc]

***CHST9* rs1436904 genetic variant contributes to prognosis of triple-negative breast cancer**

Jupeng Yuan^1,†^; Nasha Zhang^1,2,†^; Hui Zhu^2^; Jibing Liu^1^; Huaixin Xing^1^; Fei Ma^3,*^; Ming Yang^1,*^

**Authors’ affiliations:** ^1^Shandong Provincial Key Laboratory of Radiation Oncology, Cancer Research Center, Shandong Cancer Hospital affiliated to Shandong University, Shandong Academy of Medical Sciences, Jinan, Shandong Province, China; ^2^Department of Radiation Oncology, Shandong Cancer Hospital affiliated to Shandong University, Shandong Academy of Medical Sciences, Jinan, Shandong Province, China; ^3^Department of Medical Oncology, Cancer Hospital, Chinese Academy of Medical Sciences, Beijing, China.

^†^**Note:** Jupeng Yuan and Nasha Zhang contributed equally to this work.

^*^**Correspondence to:** Ming Yang, PhD, Professor, Shandong Provincial Key Laboratory of Radiation Oncology, Cancer Research Center, Shandong Cancer Hospital affiliated to Shandong University, Shandong Academy of Medical Sciences, Jinan 250117, Shandong Province, China. Tel & Fax: 86531-67626536; E-mail: aaryoung@yeah.net, yangm@sdu.edu.cn;

or,

Fei Ma, MD, Professor, Department of Medical Oncology, Cancer Hospital, Chinese Academy of Medical Sciences, Beijing 100021, China. Tel & Fax: 8610-87715711, e-mail: mafei2011@139.com.

**Supplementary Table 1.** Clinical and treatment characteristics of the TNBC patients

| Characteristics | *n* (%) |
| --- | --- |
|  | 381 |
| Age of onset (year) |  |
| Median | 49 |
| Range | 21-76 |
| BMI(Body Mass Index) |  |
| Median | 24.3 |
| Range | 16.0-39.0 |
| Breast cancer/Ovarian cancer history |  |
| Yes | 23(6.0) |
| No | 333(87.4) |
| Unknown | 25(6.6) |
| Menopausal status at diagnosis |  |
| Premenopausal | 190(49.9) |
| Postmenopausal | 168(44.1) |
| Unknown | 23(6.0) |
| Operation method |  |
| Modified radical mastectomy | 308(80.8) |
| Breast conserving surgery | 70(18.4) |
| Unknown | 3(0.8) |
| Histological type |  |
| Infiltrative nonspecific cancer | 349(91.6) |
| Others | 25(6.6) |
| Unknown | 7(1.8) |
| Histological grade |  |
| I | 108(28.3) |
| II | 198(52.0) |
| III | 60(15.8) |
| Unknown | 15(3.9) |
| Vascular invasion |  |
| Yes | 39(10.2) |
| No | 334(87.7) |
| Unknown | 8(2.1) |
| Tumor size |  |
| ≤2cm | 181(47.5) |
| >2cm | 196(51.4) |
| Unknown | 4(1.1) |
| Lymph-node involvement |  |
| Yes | 144(37.8) |
| No | 230(60.4) |
| Unknown | 7(1.8) |
| Taxane/anthracycline-based chemotherapy |  |
| Yes | 363(95.3) |
| No | 11(2.9) |
| Unknown | 7(1.8) |
| Radiotherapy |  |
| Yes | 163(42.8) |
| No | 217(56.9) |
| Unknown | 1(0.3) |

**Supplementary Table 2.** Univariate and multivariate Cox-regression analyses for DFS

| Variable | Univariate analysis | | Multivariate analysis | |
| --- | --- | --- | --- | --- |
|  | HR (95% CI) | *P* | HR (95% CI) | *P* |
| Age of onset (year) | 0.99(0.98-1.01) | 0.286 | 0.99(0.97-1.01) | 0.347 |
| BMI (Body Mass Index) | **1.04(1.00-1.09)** | **0.072** | **1.07(1.02-1.12)** | **0.003** |
| Breast cancer/Ovarian cancer history | 0.89(0.48-1.64) | 0.699 | 0.69(0.36-1.35) | 0.278 |
| Menopausal status at diagnosis | 0.82(0.60-1.10) | 0.186 | 0.92(0.59-1.44) | 0.717 |
| Operation method | 1.28(0.89-1.86) | 0.190 | 1.06(0.63-1.76) | 0.836 |
| Histological type | 1.14(0.67-1.95) | 0.623 | 1.35(0.73-2.52) | 0.337 |
| Histological grade | **1.34(1.05-1.70)** | **0.017** | 1.18(0.82-1.68) | 0.372 |
| Vascular invasion | **1.97(1.32-2.93)** | **0.001** | **1.95(1.26-3.02)** | **0.003** |
| Tumor size | 1.19(0.89-1.59) | 0.249 | 1.00(0.69-1.45) | 0.997 |
| Lymph-node involvement | **1.39(1.03-1.87)** | **0.029** | 1.06(0.68-1.66) | 0.808 |
| Taxane/anthracycline-based chemotherapy | 1.05(0.46-2.37) | 0.913 | 0.86(0.36-2.04) | 0.731 |
| Radiotherapy | **1.41(1.06-1.88)** | **0.019** | 1.42(0.91-2.22) | 0.122 |

Note: DFS, disease-free survival time; HR, hazard ratio; CI, confidence interval.

Hazard ratios (HRs) and 95% confidence intervals (CIs) for the association between clinical factors and recurrence risk was adjusted for age of onset, BMI, tumor size, lymph-node involvement, histological type, histological grade, menopausal status, vascular invasion, breast or ovarian cancer history, surgical method, taxane/anthracycline-based chemotherapy and radiotherapy.

**Supplementary Table 3.** Disease-free survival time of TNBC associated with *CHST9* rs1436904 genotypes by age of onset

| Age of onset (year) | *CHST9* rs1436904 | | | | |
| --- | --- | --- | --- | --- | --- |
|  | Genotypes | Patients  No. (%) | DFS (month)  Mean (25th, 75th) | HR (95% CI) | *P* |
| ≤49 |  | 201 |  |  |  |
|  | TT | 49(24.4) | 57.5(32, 72) | Reference |  |
|  | GT | 106(52.7) | 50.2(24, 62) | 1.76(1.02-3.04) | 0.041 |
|  | GG | 46(22.9) | 47.2(24, 69) | 1.77(0.88-3.54) | 0.109 |
|  | GT+GG | 152(75.6) | 49.3(24, 62) | 1.82(1.09-3.05) | 0.022 |
| >49 |  | 177 |  |  |  |
|  | TT | 48(27.1) | 53.1(25, 62) | Reference |  |
|  | GT | 88(49.7) | 49.9(27, 71) | 0.78(0.40-1.54) | 0.473 |
|  | GG | 41(23.2) | 46.4(28, 60) | 1.21(0.50-2.92) | 0.673 |
|  | GT+GG | 129(72.9) | 48.8(27, 65) | 1.05(0.55-2.01) | 0.888 |

Note: TNBC, Triple-negative breast cancer; DFS, disease-free survival time; HR, hazard ratio; CI, confidence interval.

Hazard ratios (HRs) and 95% confidence intervals (CIs) for the association between SNP and disease-free survival time (DFS) were estimated by Cox regression adjusted by age of onset, BMI, tumor size, lymph-node involvement, histological type, histological grade, menopausal status, vascular invasion, breast or ovarian cancer history, surgical method, taxane/anthracycline-based chemotherapy and radiotherapy.

**Supplementary** **Table 4.** Disease-free survival time of TNBC associated with *CHST9* rs1436904 genotypes by histological grade

| Histological grade | *CHST9* rs1436904 | | | | |
| --- | --- | --- | --- | --- | --- |
|  | Genotypes | Patients  No. (%) | DFS (month)  Mean (25th, 75th) | HR (95% CI) | *P* |
| I |  | 107 |  |  |  |
|  | TT | 24(22.4) | 55.3(36, 72) | Reference |  |
|  | GT | 57(53.3) | 55.5(36, 71) | 1.45(0.64-3.29) | 0.369 |
|  | GG | 26(24.3) | 51.9(24, 72) | 1.35(0.38-4.78) | 0.638 |
|  | GT+GG | 83(77.6) | 54.4(35, 71) | 1.46(0.67-3.20) | 0.340 |
| II |  | 197 |  |  |  |
|  | TT | 56(28.4) | 62.1(34, 78) | Reference |  |
|  | GT | 97(49.3) | 53.1(27, 64) | 1.84(0.98-3.47) | 0.059 |
|  | GG | 44(22.3) | 46.6(29, 60) | 2.20(0.95-5.09) | 0.065 |
|  | GT+GG | 141(71.6) | 51.0(29, 63) | 2.04(1.11-3.74) | 0.021 |
| III |  | 59 |  |  |  |
|  | TT | 16(27.1) | 32.5(12, 39) | Reference |  |
|  | GT | 30(50.9) | 34.1(12, 46) | 1.15(0.44-2.99) | 0.780 |
|  | GG | 13(22.0) | 37.5(12, 56) | 1.47(0.34-6.36) | 0.607 |
|  | GT+GG | 43(72.9) | 35.1(12, 46) | 1.33(0.57-3.10) | 0.513 |

Note: TNBC, Triple-negative breast cancer; DFS, disease-free survival time; HR, hazard ratio; CI, confidence interval.

Hazard ratios (HRs) and 95% confidence intervals (CIs) for the association between SNP and disease-free survival time (DFS) were estimated by Cox regression adjusted by age of onset, BMI, tumor size, lymph-node involvement, histological type, histological grade, menopausal status, vascular invasion, breast or ovarian cancer history, surgical method, taxane/anthracycline-based chemotherapy and radiotherapy.

**Supplementary** **Table 5.** Disease-free survival time of TNBC associated with *CHST9* rs1436904 genotypes by operation method

| Operation method | *CHST9* rs1436904 | | | | |
| --- | --- | --- | --- | --- | --- |
|  | Genotypes | Patients  No. (%) | DFS (month)  Mean (25th, 75th) | HR (95% CI) | *P* |
| Modified radical mastectomy |  | 305 |  |  |  |
|  | TT | 86(28.2) | 56.9(31, 72) | Reference |  |
|  | GT | 154(50.5) | 50.4(24, 65) | 1.38(0.89-2.13) | 0.146 |
|  | GG | 65(21.3) | 47.3(24, 62) | **1.77(1.02-3.09)** | **0.043** |
|  | GT+GG | 219(71.8) | 49.4(24, 63) | **1.52(1.01-2.29)** | **0.043** |
| Breast conserving surgery |  | 70 |  |  |  |
|  | TT | 11(15.7) | 42.5(30, 41) | Reference |  |
|  | GT | 38(54.3) | 46.4(35, 64) | 1.29(0.29-5.74) | 0.739 |
|  | GG | 21(30.0) | 46.3(24, 64) | 2.09(0.38-11.40) | 0.395 |
|  | GT+GG | 59(84.3) | 46.4(24, 64) | 1.40(0.38-5.09) | 0.613 |

Note: TNBC, Triple-negative breast cancer; DFS, disease-free survival time; HR, hazard ratio; CI, confidence interval.

Hazard ratios (HRs) and 95% confidence intervals (CIs) for the association between SNP and disease-free survival time (DFS) were estimated by Cox regression adjusted by age of onset, BMI, tumor size, lymph-node involvement, histological type, histological grade, menopausal status, vascular invasion, breast or ovarian cancer history, surgical method, taxane/anthracycline-based chemotherapy and radiotherapy.

**Supplementary** **Table 6.** Disease-free survival time of TNBC associated with *CHST9* rs1436904 genotypes by BMI

| BMI | *CHST9* rs1436904 | | | | |
| --- | --- | --- | --- | --- | --- |
|  | Genotypes | Patients  No. (%) | DFS (month)  Mean (25th, 75th) | HR (95% CI) | *P* |
| ≤24.3 |  | 174 |  |  |  |
|  | TT | 44(25.3) | 58.4(36, 68) | Reference |  |
|  | GT | 89(51.1) | 50.4(24, 67) | 1.04(0.54-2.03) | 0.903 |
|  | GG | 41(23.6) | 45.0(24, 60) | 2.29(1.06-4.98) | 0.036 |
|  | GT+GG | 130(74.7) | 48.7(24, 67) | 1.47(0.79-2.74) | 0.228 |
| >24.3 |  | 177 |  |  |  |
|  | TT | 49(27.7) | 53.9(25, 72) | Reference |  |
|  | GT | 89(50.3) | 49.7(27, 61) | 1.68(0.96-2.94) | 0.071 |
|  | GG | 39(22.0) | 48.4(32, 62) | 1.61(0.76-3.42) | 0.212 |
|  | GT+GG | 128(72.3) | 49.3(28, 62) | 1.59(0.94-2.68) | 0.085 |

Note: TNBC, Triple-negative breast cancer; DFS, disease-free survival time; HR, hazard ratio; CI, confidence interval.

Hazard ratios (HRs) and 95% confidence intervals (CIs) for the association between SNP and disease-free survival time (DFS) were estimated by Cox regression adjusted by age of onset, BMI, tumor size, lymph-node involvement, histological type, histological grade, menopausal status, vascular invasion, breast or ovarian cancer history, surgical method, taxane/anthracycline-based chemotherapy and radiotherapy.

**Supplementary** **Table 7.** Disease-free survival time of TNBC associated with *CHST9* rs1436904 genotypes by radiotherapy

| Radiotherapy | *CHST9* rs1436904 | | | | |
| --- | --- | --- | --- | --- | --- |
|  | Genotypes | Patients  No. (%) | DFS (month)  Mean (25th, 75th) | HR (95% CI) | *P* |
| No |  | 216 |  |  |  |
|  | TT | 56(25.9) | 60.4(36, 72) | Reference |  |
|  | GT | 116(53.7) | 52.6(27, 71) | 1.50(0.85-2.65) | 0.160 |
|  | GG | 44(20.4) | 54.0(32, 71) | 1.49(0.71-3.14) | 0.292 |
|  | GT+GG | 160(74.1) | 53.0(30, 71) | 1.45(0.84-2.49) | 0.179 |
| Yes |  | 161 |  |  |  |
|  | TT | 41(25.5) | 48.4(24, 54) | Reference |  |
|  | GT | 78(48.4) | 46.3(24, 61) | 1.35(0.68-2.69) | 0.395 |
|  | GG | 42(26.1) | 39.8(24, 48) | **2.19(1.07-4.49)** | **0.033** |
|  | GT+GG | 120(74.5) | 44.0(24, 60) | 1.71(0.93-3.14) | 0.086 |

Note: TNBC, Triple-negative breast cancer; DFS, disease-free survival time; HR, hazard ratio; CI, confidence interval.

Hazard ratios (HRs) and 95% confidence intervals (CIs) for the association between SNP and disease-free survival time (DFS) were estimated by Cox regression adjusted by age of onset, BMI, tumor size, lymph-node involvement, histological type, histological grade, menopausal status, vascular invasion, breast or ovarian cancer history, surgical method, taxane/anthracycline-based chemotherapy and radiotherapy.
